# Supplementary material for: Association between Lower Intake of Minerals and Depressive Symptoms among Elderly Japanese Women but Not Men: Findings from Shika Study
Source: Nutrients. 2019 Feb 13;11(2):389. doi: 10.3390/nu11020389 (PMC6412241; doi:10.3390/nu11020389)
Supplement: Supplementary file 1 [file nutrients-11-00389-s001.pdf]

Association between Lower Intake of Minerals and Depressive Symptoms among Elderly Japanese Women, but Not Men: Findings from Shika Study

Thao Thi Thu Nguyen*, Sakae Miyagi, Hiromasa Tsujiguchi, Yasuhiro Kambayashi, Akinori Hara, Haruki Nakamura, Keita Suzuki, Yohei Yamada, Yukari Shimizu, and Hiroyuki Nakamura

Supplementary

Table S1: The percentiles of mineral intake

Mineral	All population (n=1423)			MALE (n=653)			FEMALE (n=770)		
	25th percentile	50th percentile	75th percentile	25th percentile	50th percentile	75th percentile	25th percentile	50th percentile	75th percentile
Sodium	2.12	2.44	2.78	2.09	2.43	2.79	2.15	2.45	2.78
Potassium	1.16	1.40	1.70	1.09	1.30	1.57	1.23	1.49	1.82
Calcium	238.98	314.42	387.83	225.57	299.08	364.14	253.98	333.93	411.43
Magnesium	120.64	140.50	164.80	118.24	135.92	157.43	122.50	144.76	170.07
Phosphorus	505.67	585.43	672.83	491.00	564.22	649.87	523.73	603.47	698.72
Iron	3.60	4.33	5.13	3.51	4.12	4.91	3.73	4.47	5.30
Zinc	4.07	4.50	4.91	4.00	4.36	4.81	4.18	4.59	5.03
Copper	0.59	0.65	0.73	0.57	0.63	0.71	0.61	0.67	0.74
Manganese	1.34	1.58	1.91	1.32	1.56	1.91	1.36	1.59	1.91

Table S2: Odd ratios and 95% CI for depressive symptoms across mineral intake

Minerals	Crude			Full-adjusted model¶		
	B	OR (95% CI)	p-value	B	OR (95% CI)	p-value
Sodium	−0.149	0.861 (0.675–1.099)	0.229	−0.241	0.786 (0.607–1.018)	0.068
Potassium	−0.678	0.508 (0.367–0.702)	<0.001	−0.602	0.548 (0.388–0.773)	0.001
Calcium	−0.001	0.999 (0.997–1.000)	0.015	−0.001	0.999 (0.998–1.000)	0.089
Magnesium	−0.009	0.991 (0.987–0.995)	<0.001	−0.007	0.993 (0.989–0.997)	0.001
Phosphorus	−0.001	0.999 (0.998–1.000)	0.019	−0.001	0.999 (0.998–1.000)	0.144
Iron	−0.208	0.812 (0.722–0.914)	0.001	−0.202	0.817 (0.722–0.925)	0.001
Zinc	−0.255	0.775 (0.634–0.946)	0.012	−0.271	0.762 (0.609–0.954)	0.018
Copper	−1.515	0.220 (0.067–0.724)	0.013	−2.206	0.110 (0.029–0.419)	0.001
Manganese	−0.115	0.892 (0.678–1.172)	0.411	−0.249	0.780 (0.585–1.038)	0.088

B: coefficient, OR: odd ratio, CI: confidence interval

¶adjusted for age, BMI, living status, having a job status, married status, smoking status, drinking alcohol, alcohol consumption, total energy, hypertension, diabetes, hyperlipidemia

Table S3: Odd ratios and 95% CI for depressive symptoms across mineral intake levels

Minerals		Crude		Full–adjusted model¶	
		β	OR (95%CI)	β	OR (95%CI)
Sodium	Quartile 1		1.000 (ref)		1.000 (ref)
	Quartile 2	−0.279	0.757 (0.537–1.068)	−0.506	0.603 (0.407–0.893)*
	Quartile 3	−0.210	0.810 (0.577–1.139)	−0.195	0.823 (0.566–1.197)
	Quartile 4	−0.072	0.930 (0.667–1.298)	−0.302	0.739 (0.502–1.088)
	p−value		0.370		0.084
Potassium	Quartile 1		1.000 (ref)		1.000 (ref)
	Quartile 2	−0.092	0.912 (0.664–1.253)	0.008	1.008 (0.704–1.442)
	Quartile 3	−0.501	0.606 (0.432–0.851)*	−0.396	0.673 (0.460–0.985)*
	Quartile 4	−0.842	0.431 (0.299–0.620)**	−0.817	0.442 (0.290–0.675)**
	p−value		<0.001		<0.001
Calcium	Quartile 1		1.000 (ref)		1.000 (ref)
	Quartile 2	−0.167	0.846 (0.610–1.174)	−0.023	0.977 (0.676–1.413)
	Quartile 3	−0.457	0.633 (0.449–0.893)*	−0.454	0.635 (0.428–0.943)*
	Quartile 4	−0.402	0.669 (0.476–0.940)*	−0.263	0.769 (0.523–1.130)
	p−value		0.030		0.083
Magnesium	Quartile 1		1.000 (ref)		1.000 (ref)
	Quartile 2	−0.399	0.671 (0.484–0.931)	−0.211	0.810 (0.563–1.164)
	Quartile 3	−0.449	0.638 (0.459–0.888)*	−0.348	0.706 (0.486–1.025)
	Quartile 4	−0.801	0.449 (0.315–0.639)**	−0.620	0.538 (0.361–0.802)*
	p−value		<0.001		0.021
Phosphorus	Quartile 1		1.000 (ref)		1.000 (ref)
	Quartile 2	−0.217	0.805 (0.577–1.124)	−0.179	0.836 (0.575–1.215)
	Quartile 3	−0.189	0.827 (0.594–1.154)	−0.159	0.853 (0.583–1.247)
	Quartile 4	−0.449	0.639 (0.451–0.904)*	−0.301	0.740 (0.498–1.100)
	p−value		0.093		0.515
Iron	Quartile 1		1.000 (ref)		1.000 (ref)
	Quartile 2	−0.215	0.806 (0.578–1.125)	−0.186	0.831 (0.570–1.211)

	Quartile 3	−0.158	0.854 (0.614–1.187)	−0.092	0.912 (0.629–1.324)
	Quartile 4	−0.554	0.575 (0.403–0.818)*	−0.562	0.570 (0.382–0.851)*
	p−value		0.022		0.040
Zinc	Quartile 1		1.000 (ref)		1.000 (ref)
	Quartile 2	−0.088	0.916 (0.655–1.281)	0.027	1.028 (0.699–1.510)
	Quartile 3	0.094	1.099 (0.792–1.523)	0.125	1.133 (0.775–1.657)
	Quartile 4	−0.576	0.562 (0.390–0.811)*	−0.536	0.585 (0.382–896)*
	p−value		0.002		0.010
Copper	Quartile 1		1.000 (ref)		1.000 (ref)
	Quartile 2	−0.299	0.741 (0.532–1.033)	−0.270	0.763 (0.520–1.120)
	Quartile 3	−0.272	0.762 (0.548–1.061)	−0.280	0.756 (0.515–1.110)
	Quartile 4	−0.570	0.566 (0.399–0.802)*	−0.640	0.527 (0.350–0.793)*
	p−value		0.015		0.024
Manganese	Quartile 1		1.000 (ref)		1.000 (ref)
	Quartile 2	0.056	1.058 (0.755–1.483)	0.041	1.042 (0.711–1.529)
	Quartile 3	0.030	1.030 (0.734–1.447)	−0.015	0.985 (0.671–1.445)
	Quartile 4	−0.175	0.839 (0.591–1.191)	−0.274	0.760 (0.510–1.133)
	p−value		0.567		0.389

B: coefficient, OR: odd ratio, CI: confidence interval

¶adjusted for age, BMI, living status, having a job status, married status, smoking status, alcohol consumption, total energy, hypertension, diabetes, hyperlipidemia; *p<0.05, **p<0.001
